# Silver Nanoparticle-Coated Polyhydroxyalkanoate Based Electrospun Fibers for Wound Dressing Applications

**DOI:** 10.3390/ma14174907

**Published:** 2021-08-28

**Authors:** Ozlem Ipek Kalaoglu-Altan, Havva Baskan, Timo Meireman, Pooja Basnett, Bahareh Azimi, Alessandra Fusco, Niccola Funel, Giovanna Donnarumma, Andrea Lazzeri, Ipsita Roy, Serena Danti, Karen De Clerck

**Affiliations:** 1 Department of Materials, Textiles and Chemical Engineering, Ghent University, 9000 Ghent, Belgium; kalaoglualtan@itu.edu.tr (O.I.K.-A.); baskan@itu.edu.tr (H.B.); timo.meireman@ugent.be (T.M.); 2 Department of Textile Engineering, Istanbul Technical University, 34437 Istanbul, Turkey; 3 School of Life Sciences, College of Liberal Arts and Sciences, University of Westminster, London W1W 6UW, UK; p.basnett@westminster.ac.uk; 4 Interuniversity National Consortiums of Materials Science and Technology (INSTM), 50121 Firenze, Italy; bahareh.azimi@ing.unipi.it (B.A.); alessandra.fusco@unicampania.it (A.F.); giovanna.donnarumma@unicampania.it (G.D.); 5 Department of Civil and Industrial Engineering, University of Pisa, 56122 Pisa, Italy; andrea.lazzeri@unipi.it (A.L.); serena.danti@unipi.it (S.D.); 6 Department of Experimental Medicine, University of Campania “Luigi Vanvitelli”, 80125 Naples, Italy; 7 Department of Translational Research and New Technologies in Medicine and Surgery, University of Pisa, 56126 Pisa, Italy; niccola.funel@gmail.com; 8 Department of Materials Science and Engineering, Faculty of Engineering, University of Sheffield, Sheffield S3 7HQ, UK; i.roy@sheffield.ac.uk

**Keywords:** polyhydroxyalkanoates, eco-friendly nanofibers, wound dressings, low-impact biomedical applications, immunomodulation, defensin, keratinocytes

## Abstract

Wound dressings are high performance and high value products which can improve the regeneration of damaged skin. In these products, bioresorption and biocompatibility play a key role. The aim of this study is to provide progress in this area via nanofabrication and antimicrobial natural materials. Polyhydroxyalkanoates (PHAs) are a bio-based family of polymers that possess high biocompatibility and skin regenerative properties. In this study, a blend of poly(3-hydroxybutyrate) (P(3HB)) and poly(3-hydroxyoctanoate-*co*-3-hydroxy decanoate) (P(3HO-*co*-3HD)) was electrospun into P(3HB))/P(3HO-*co*-3HD) nanofibers to obtain materials with a high surface area and good handling performance. The nanofibers were then modified with silver nanoparticles (AgNPs) via the dip-coating method. The silver-containing nanofiber meshes showed good cytocompatibility and interesting immunomodulatory properties in vitro, together with the capability of stimulating the human beta defensin 2 and cytokeratin expression in human keratinocytes (HaCaT cells), which makes them promising materials for wound dressing applications.

## 1. Introduction

Nanofibers have received much attention in biomedical applications lately. These materials have a high surface area, porosity, versatility in material choice and morphological similarities to the naturally occurring extracellular matrix, which enhances cell attachment and proliferation, transport of nutrients, gas exchange and waste excretion. Furthermore, the physical and/or chemical properties of the electrospun nanofibers can be modified by encapsulation and or immobilization of bio-active species to receive specific biological reactions [1]. The unique properties of nanofibers have attracted particular attention for wound healing applications. Contemporary wound dressings should accelerate healing with a pleasing aesthetic result [2,3]. For this purpose, they should be hemostatic, absorb exudates, manage moisture balance and thermal insulation, protect against infections and be low cost. Researchers have shown that nanofibers which function well as wound dressings have good hemostasis, absorbability and semi-permeability [2,4]. Moreover, multifunctional dressings can easily be designed by the incorporation of multiple therapeutic compounds.

Nanofibers are most frequently produced by the electrospinning technique, as it is an inexpensive and reliable method to create fibers with diameters ranging from the nanometer scale to a few micrometers [5,6]. Many different natural and synthetic polymers have been electrospun for applications such as tissue engineering, wound dressing and drug delivery [1,7,8,9,10,11]. For such applications, biodegradable and biocompatible polymers are more favorable over others, as they metabolize into biocompatible degradation products in the human body.

Polyhydroxyalkanoates (PHAs) are fully biodegradable polymers which are biosynthesized with high purity by a wide range of Gram-positive and Gram-negative bacteria from inexpensive waste carbon sources [12,13,14,15,16,17]. Moreover, they are biocompatible, non-toxic, insoluble in water, piezoelectric, thermoplastic and/or elastomeric and available at an industrial scale. These properties make them suitable for various applications such as the packaging industry, medicine, pharmacy, agriculture, the food industry, and the production of paints. Nowadays, PHAs are extensively investigated for their use in biomedical applications, including drug delivery, wound healing, vascular grafting, orthopedic applications, and urological stents [18,19,20]. PHAs with 3–5 carbon atoms such as poly(3-hydroxybutyrate) (P(3HB)) and poly(4-hydroxybutyrate) (P(4HB)) are considered as short chain length PHAs (scl-PHAs). Medium chain length PHAs (mcl-PHAs) contain 6–14 carbon atoms, which include homopolymers such as poly(3-hydroxyhexanoate), P(3HHx), poly(3-hydroxyoctanoate), P(3HO) and heteropolymers such as P(3HHx-*co*-3HO). Scl-PHAs typically have a higher molecular weight, crystallinity and brittleness, while mcl-PHAs are more elastomeric. Mcl-PHAs and their copolymers are preferred for biomedical applications where flexible biomaterials are required, such as heart valves or controlled drug delivery [21,22]. The structural diversity of mcl-PHAs is higher than scl-PHAs, so they can be easily designed according to what is needed, as the monomer content of PHAs affects their physical and chemical characteristics. However, one drawback of the mcl-PHAs for electrospinning is their low glass transition temperature (T_g_), which limits their spinnability and causes sticky and fused fibers. Several groups have investigated the electrospinning of different scl-PHAs, including P(3HB), poly(3-hydroxybutyrate-*co*-3-hydroxyvalerate) P(3HB-*co*-3HV), poly(3-hydroxybutyrate-*co*-4-hydroxybutyrate) (P3HB-*co*-4HB), poly(3-hydroxybutyrate-*co*-3-hydroxyhexanoate) P(HB-*co*-3HHx) and poly(3-hydroxybutyrate-*co*-3-hydroxyvalerate-*co*-3-hydroxyhexanoate) P(3HB-*co*-3HV-*co*-3HHx) [23,24,25,26,27,28,29]. Recent examples by Liu and coworkers introduced both single-nozzle and coaxial electrospinning of mcl-PHA poly (3-hydroxyoctanoate-*co*-3-hydroxyhexanoate) P(3HO-*co*-3HHx) nanofibers by blending with a scl-PHA, P(3HB-*co*-3HV) [30,31]. Electrospun PHA fibrous meshes can be surface-functionalized with micro-/nanoparticles to introduce specific functionalities, such as chitin nanofibrils, useful in skin regeneration and repair [32].

In recent years, metal nanoparticle-containing electrospun nanofibers have attained widespread attraction for various applications such as bio/gas sensing, catalysis, and antimicrobial wound dressings [33,34]. Among many metal nanoparticles and their metal oxides, such as copper, gold, ZnO, CuO and TiO_2_, silver nanoparticles (AgNPs) have been investigated most intensively in the medical field due to their excellent chemical stability, high conductivity, catalytic activity, localized surface plasma resonance as well as their antibacterial and anti-inflammatory properties and efficacy against a broad range of bacteria found in chronic wounds [35,36]. AgNPs show lower toxicity compared to conventional silver compounds [37,38]. Several studies on the combination of silver nanoparticles with PHAs have been performed by Lagaron and coworkers including the production of AgNPs by chemical reduction in P(3HB-*co*-3HV) suspensions [39], in situ stabilized nanosilver-based antimicrobial P(3HB-*co*-3HV) nanocomposites [40] and an antimicrobial active multilayer system composed of a P(3HB-*co*-3HV) film coated with P(3HB-*co*-3HV)/AgNPs nanofibers [41], in which silver nitrate was chemically reduced to generate AgNPs.

Antimicrobial biomaterials are very useful in a number of biomedical applications related to many diseases, especially those concerning or deriving from skin damage [42,43]. Some types of wounds cannot self-heal completely, often due to the presence of concurrent pathologies. Therefore, they tend to become chronic via self-sustaining inflammatory processes, which degrade the growth factors deputed to healing, thus leading to recurrent infections. In those cases, the application of specific biomaterial dressings able to stimulate and/or accelerate the wound healing process can be of great importance to restore the normal native tissues. For this reason, the search for highly biocompatible and antibacterial materials able to modulate the immune reaction in a successful way is essential, and electrospun nanofibers are gaining popularity in this field.

The biological mediators of innate immunity and inflammation are cytokines, biological molecules that act as soluble mediators of natural immunity and the immune response [44]. The most frequently studied cytokines are several proinflammatory cytokines, interleukin 1 (IL 1), tumor necrosis factor α (TNF-α), interleukin 6 (IL-6), interleukin 8 (IL-8), and the anti-inflammatory cytokine transforming growth factor β (TGF-β). In response to irritant products and injuries the epidermal keratinocytes also secrete antimicrobial peptides. Human β-defensin 2 (HBD-2) acts as an endogenous antibiotic against bacteria, fungi and the envelope of some viruses, and can be activated as an innate immune response by endogenous stimuli, infections or wounds [45]. Inflammation is essential to initiate the wound repair, but it must be modulated over time to achieve a normal uncomplicated healing. Therefore, new bioactive materials, which are able to stimulate intrinsic antimicrobial activity of cells while modulating the inflammatory process, are interesting candidates as wound dressings. In addition, an improved coating method based on AgNPs can be promising for reducing the toxicity while improving the antimicrobial activity.

The goal of this paper was to design nanofibrous wound dressings from bio-based polymers (i.e., PHAs) in order to contribute to reducing environmental pollution and to provide antimicrobial features to the PHA membranes using AgNPs. For the production of nanofibers with silver nanoparticles, there have been suggested some chemical and physical methods such as *in-situ* reduction of silver nanoparticle precursor (generally silver nitrate) to silver nanoparticles in a polymer solution [46,47,48], application of UV light to nanofibers that contain silver nanoparticle precursor [49,50], etc. In *in-situ* reduction process, *N,N* dimethylformamide (DMF) is used as a reductant, which is a very harmful solvent, and the process itself takes a long time, approximately 5–10 days. Moreover, it is not possible to achieve a 100% successful reduction of silver precursor to silver nanoparticles. In addition, in the physical reduction processes, UV-light is used and it is not a very cheap technique in terms of the consumed energy and time. However, dip-coating method enables the obtaining of silver nanoparticle containing nanofibers via a simple, cheap, environmentally friendly technique for biomedical applications. To the best of our knowledge, PHA-based nanofibers were not functionalized with silver nanofibers via dip-coating. For this purpose, we proposed an alternative method for AgNP deposition via dip-coating technique using a colloidal silver solution that allows a timesaving, simple and reagent-free way to produce antimicrobial P(3HB)/P(3HO-*co*-3HD) membranes. We first examined the electrospinning of elastomeric P(3HO-*co*-3HD) using an eco-friendly solvent mixture, but wet fibers were obtained due to the low T_g_ of the polymer. In order to tune the electrospinnability, elasticity and mechanical properties, we prepared blends of the elastomeric mcl-PHA, P(3HO-*co*-3HD) with scl-PHA, P(3HB) which has a higher T_g_ and brittleness. Since scl-P(3HB) could not be dissolved with the previously used eco-friendly solvent system, a chlorinated solvent mixture was used for the electrospinning of P(3HB)/P(3HO-*co*-3HD) blends. The electrospun P(3HB)/P(3HO-*co*-3HD) nanofibers were then modified with AgNPs via a dip-coating method. The antimicrobial properties of the modified nanofibers were investigated and were found to be promising for wound dressing applications. Conventional wound dressings such as acrylic, polyethylene or polypropylene-based pads usually suffer from a lack of recycling—they are either incinerated or landfilled. In our study, the bio-based material of the nanofibrous mats offer organic composting as an option for waste management. Moreover, the high polarity of the PHAs and the porosity of the fibrous meshes will provide advantages such as easy and proper impregnation of the agents, odor suppression, efficacy of treatment and easy removal of the dressing from the skin. Having a properly functional bioactive and biocompatible wound dressing which combines a reduced environmental impact with remarkable antibacterial properties would greatly improve the portfolio of wound dressing products.

## 2. Materials and Methods

The P(3HO-*co*-3HD) was created within 15 L bioreactors (Applikon Biotechnology, Tewkesbury, UK) containing a 10 L mixture of *Pseudomonas mendocina* CH50 and 20 g/L glucose. Subsequently, a two-stage batch fermentation was applied such as described in Basnett et al., 2020 [51]. The production of P(3HB) was carried out with a similar protocol. However, this time *Bacillus subtilis* OK2 was used with single-stage batch fermentation. The P(3HB) produced exhibited a M_w_ of 796 ±40 kDa, 2–4% elongation at break, T_g_ = 2.9 °C, and the mcl-PHA P(3HO-*co*-3HD) exhibited a Mw of 350 ± 60 kDa, 440% elongation at break, and T_g_ between 48 °C and 44 °C. A specific purification protocol was applied to obtain ultra-pure PHAs as described in Basnett et al., 2020. Aqueous colloidal silver solution (4000 ppm, size of the AgNPs in the solution was between 10 and 80 nm) was purchased from Argenol Laboratories (Zaragoza, Spain). Lithium bromide (LiBr), ethanol (EtOH), and Dulbecco’s phosphate-buffered saline were purchased from Sigma-Aldrich (Saint Louis, MO, USA). The HaCaT cell line was provided by CLS-Cell Lines Service, Eppenheim, Germany. Dulbecco’s Modified Essential Medium (DMEM), L-glutamine, MgCL2, penicillin, streptomycin (penstrep) and fetal calf serum were bought from Invitrogen (Carlsbad, CA, USA). Alamar Blue was obtained from Thermo Fisher Scientific (Waltham, MA, USA). LC Fast Start DNA Master SYBR Green kit was provided by Roche Applied Science (Basel, Switzerland). The solvents used for solubility studies and electrospinning include acetone, anisole, 2-butanol, butyl acetate, chloroform, dichloromethane, diethyl ether, dimethyl carbonate, ethyl acetate, and methyl ethyl ketone, which were purchased from Sigma-Aldrich and have a purity above 98%. PanCK monoclonal antibody, hydrogen peroxidase, citrate buffer, standard avidin–biotin conjugated complex and DAB staining were provided by Dako (from Agilent; Carpinteria, CA, USA). Formalin was bought from Bio-Optica (Milan, Italy).

The nanofibers were analyzed morphologically by an FEI Quanta 200 F FE-SEM and a Phenom ProX SEM (Waltham, MA, USA). The samples were coated with gold using a sputter coater (Balzer Union SKD030, Balzers, Liechtenstein) prior to analysis. The nanofiber diameters were calculated via ImageJ software (1.51v). Additionally, the histogram graphs of the nanofibers were obtained from the measured diameter values by using the Origin software (Origin 2017). The average nanofiber diameter and the standard deviation were based on 50 measurements per sample. In addition, the weight percentage (wt%) and the atomic percentage of silver nanoparticles on the silver nanoparticle-coated samples were determined by an FEI Quanta 200F FE-SEM-EDX. An attenuated total reflectance Fourier transform infrared (ATR-FTIR) spectrometer (Nicolet iS50, Waltham, MA, USA) from Thermo Scientific was used to record the IR spectra of the samples. The spectra were collected after 32 scans for each sample between 4000 cm^−1^ and 400 cm^−1^ with a wavenumber resolution of 4 cm^−1^. Ultraviolet–visible spectroscopy of the nanofiber samples was examined by a Perkin Elmer Lambda 900 UV-Vis spectrophotometer in reflection using an integrated sphere (Spectralon Labsphere 150 mm, New Hampshire, USA). Spectra were recorded in the range of 200–800 nm with a reflection data interval of 4 nm. The obtained reflection was converted into Kubelka–Munk (K–M). Electrospinning of P(3HO-*co*-3HD) was performed in a climate chamber (Weiss WK-340/40) with temperature of 10–40 °C and relative humidity (RH) of 10–40%. The nanofibrous membranes were dip-coated in an aqueous colloidal silver solution at room temperature. This coating was performed at a speed of 170 mm/min with the aid of computer-regulated dip-coating equipment (KSV Instruments). The entire coating process happened in a clean room (class 100,000/1000). The samples were dip-coated in a silver solution (4000 ppm) by immersion for 1 min 3 successive times.

### 2.1. Optimization of Electrospinning Parameters for P(3HO-co-3HD) Nanofibers and Electrospinning of P(3HB)/P(3HO-co-3HD) Nanofibers

P(3HO-*co*-3HD) was electrospun in a solvent mixture containing anisole (30%, *v*/*v*), acetone (35%, *v*/*v*) and ether (35%, *v*/*v*) with a flow rate of 0.5 mL/h, tip-to-collector distance of 40 cm and voltage of 40 kV in a humidity (relative humidity of 10–40%) and temperature-controlled (10–40 °C) climate chamber. A quantity of 0.002 g/mL LiBr was added into the electrospinning solution to increase the conductivity.

Since P(3HO-*co*-3HD) is elastomeric and has a low T_g_, which creates difficulties in spinning such as fused fibers, it was blended with P(3HB) that has higher T_g_ and tends to produce fibers with better morphology.

Polymeric solutions at 11 wt% and 5.5 wt% of P(3HB)/P(3HO-*co*-3HD) (1/10, wt/wt) and a 5 wt% of P(3HB)/P(3HO-*co*-3HD) (1/3, *w*/*w*) were prepared in a chloroform/2-butanol (70/30, *v*/*v*) solvent system and stirred for 24 h at room temperature. A quantity of 0.002 g/mL LiBr was added into the electrospinning solution to increase the conductivity. The relative humidity (RH) was 38 ± 2.1% and the temperature was 22 ± 1.6 °C during the electrospinning process for the 5 wt% of P(3HB)/P(3HO-*co*-3HD) solution.

For the P(3HB)/P(3HO-*co*-3HD) (1/10, *w*/*w*) polymer solutions, a voltage of 40 kV, a 40 cm tip-to-collector distance and a flow rate of 0.5 mL/h was used. Nanofibers were collected on a chitosan non-woven carrier that was backed by an aluminum foil.

The drawback of P(3HB) is its brittleness. As the aim was to produce nanofibers with good morphology without losing the elasticity, the ratio of P(3HB)/P(3HO-*co*-3HD) was selected as 1/3 (*w*/*w*), which was suitable to produce uniform and continuous fibers with low diameters that were also not brittle.

For the P(3HB)/P(3HO-*co*-3HD) (1/3, *w*/*w*) solution, electrospinning parameters of 20 kV applied voltage, 15 cm distance between the needle tip and the collector, 1 mL/h feed-rate were applied, and nanofibers were collected on a flat collector which was covered by aluminum foil.

During the electrospinning process, in order to minimize fiber fused morphologies due to an insufficient evaporation of the solvent, the tip-to-collector distance had to be larger than 10 cm. Thus, for all electrospinning experiments, the distances between the needle tip-to collector were varied in the range of 15–40 cm.

### 2.2. Silver Deposition on P(3HB)/P(3HO-co-3HD) Nanofibers

The P(3HB)/P(3HO-*co*-3HD) nanofibers were modified with an aqueous colloidal silver solution (4000 ppm) via a dip-coating technique. The size of the nanoparticles in the solution ranged between 10 and 80 nm. For dip-coating, the nanofibrous scaffolds with a size of 30 mm × 60 mm were prepared, clamped from two sides and hung on the portable holder of the dip-coater. The samples were dipped into the colloidal silver solution and withdrawn with a speed of 170 mm/min. Three different protocols were tried, including dipping and withdrawing immediately, dipping and withdrawing after 1 min and dipping and withdrawing 3 successive times. In all cases, the withdrawing speed was the same and the samples were washed with distilled water and dried.

### 2.3. Biological Evaluation of P(3HB)/P(3HO-co-3HD) Nanofibers

Blank and AgNP-containing P(3HB)/P(3HO-*co*-3HD) nanofibers have been sterilized in EtOH overnight, then DPBS was used to rinse the nanofibers three times before usage. DMEM was used to culture the cell line HaCaT combined with 1% Penstrep, 1% glutamine and 10% serum of calf fetuses at 37 °C, combined with 5% CO_2_ and humidified air. The cells were plated on the glass slides coated with the fibrous samples and cultured for 6 h and 24 h.

To perform Alamar Blue assay, the HaCaT cells at a confluence of 80% have been incubated with the samples for a duration of 24 h. At the endpoint, 0.5 mg/mL resazurine was included, followed by another 4 h incubation to enable the metabolic reaction. The supernatants’ absorbance (λ) was then determined with the aid of a spectrophotometer (Victor3; Perkin Elmer, Waltham, MA, USA) and a reading at two wavelengths (i.e., 570 nm and 600 nm). At the end, the relative reduction of Alamar Blue (%ABred) was determined by associating the absorbance and the dye’s molar extinction coefficients at the aforementioned wavelengths, following the protocol suggested by the manufacturer. The obtained values are entered in the equation below, in which: c = control, s = sample, and λ = absorbance:

(1) $$\%{\mathrm{AB}}_{\mathrm{red}}=100\cdot \frac{\left(117,216\cdot \lambda_{s\left(570\;nm\right)}-\;80,586\cdot \lambda_{s\left(600\;nm\right)}\right)}{\left(155,677\cdot \lambda_{c\left(600\;nm\right)}-\;14,652\cdot \lambda_{c\left(570\;nm\right)}\right)}$$

The results obtained are expressed as %ABred, which is related to metabolically active cells.

The skin immunomodulatory properties of Ag-loaded and plain P(3HB)/P(3HO-*co*-3HD) nanofibers were determined. The HaCaT cells were seeded within 12-well plates up to a confluence of 80%; therefore, they were incubated for 6 h and 24 h with the glass slides coated by the nanofibers (*n* = 3). At these respective moments, all the RNA was isolated with the aid of Trizol. Subsequently, 1 µM of RNA was reverse-transcribed into the complementary DNA (cDNA). This was carried out with the aid of random hexamer primers at 42 °C for a duration of 45 min such as stated within the guidelines of the manufacturer. A LC FAST START DNA Master SYBR Green kit was used to carry out the quantitative reverse transcriptase polymer chain reaction (qRT-PCR). A quantity of 10 ng of total RNA and 2 µL of cDNA were used in a final volume of 20 µL with 3 mM MgCl2 and 0.5 µM sense and antisense primers, such as depicted in Table 1 [32,33]. The expression of TNF-α, TGF-β and HBD-2 as well as the interleukins IL-1α, IL-1β, IL-6 and IL-8 was evaluated with qRT-PCR.

Finally, the glass slides covered by the nanofibers were seeded with HaCaT cells and their capability of expressing cytokeratin was evaluated via immunocytochemistry (IHC). The antigenicity was retrieved by treating the slides at 100 °C in a steamer with 10 mMol citrate buffer (pH 6.0) for 60 min. Subsequently, the sections were immersed for 20 min in a methanol solution with 0.3% hydrogen peroxidase to stop the activity of the endogenous peroxidase. Afterwards, nonspecific binding was reduced by incubation in a 2.5% blocking serum. The specimens were incubated for 90 min at 37 °C with the primary antibody PanCK. Both the processes of the standard avidin–biotin conjugated complexing and the subsequent DAB staining were automated according to the surgical pathology guidelines (BenchmarkDX; Ventana Systems, Harvard, MA, USA). The immunopositive epithelial cells were highlighted by the brown precipitate on the cell membranes and in their cytoplasm. Negative controls were obtained by subtraction of primary antibody during the standard IHC procedure.

## 3. Results and Discussion

### 3.1. Eco-Friendly Electrospinning of P(3HO-co-3HD)

**Figure 1:**
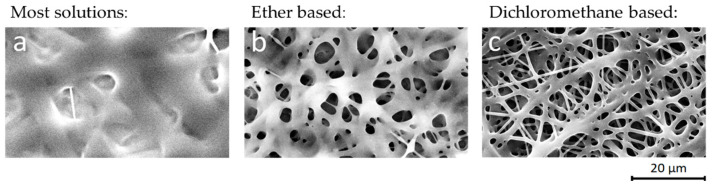
Electrospinning P(3HO-*co*-3HD) with ethyl acetate (**a**). Solutions based on green solvents (such as ethyl acetate, butyl acetate, dimethyl carbonate, methyl ethyl ketone) mostly result in bad quality fiber depositions with the nanofibers being fused together. Fast evaporating solvents such as diethyl ether result in porous membranes (**b**), but still the porosity is reduced. Fast evaporating halogenated solvents such as dichloromethane (**c**) and chloroform result in a porous nanofiber structure.

The non-toxic biosourced poly(3-hydroxyoctanoate-*co*-3-hydroxydecanoate) P(3HO-*co*-3HD) is a biocompatible and biodegradable polymer with high ductility (440% elongation at break), which makes it an ideal candidate for body contact medical applications such as wound dressings [19]. To a large extent, the polymer owes its flexibility and low T_g_ (below −40 °C) to its medium chain length alkane side-groups and its copolymeric nature [23]. These properties also contribute to the high solubility (>10 wt%) at room temperature we found for a wide range of eco-friendly solvents [52,53], including acetone, anisole, butyl acetate, diethyl ether, dimethyl carbonate, ethyl acetate, and methyl ethyl ketone. However, electrospinning of these solutions based on green solvents mostly results in bad quality fiber depositions with wet and fused nanofibers and reduced porosity (Figure 1a,b). The non-ecofriendly but fast evaporating solvents chloroform and dichloromethane result in a more porous nanofibrous membrane, although the fibers are still fused together, such as is shown in Figure 1c.

**Figure 2:**
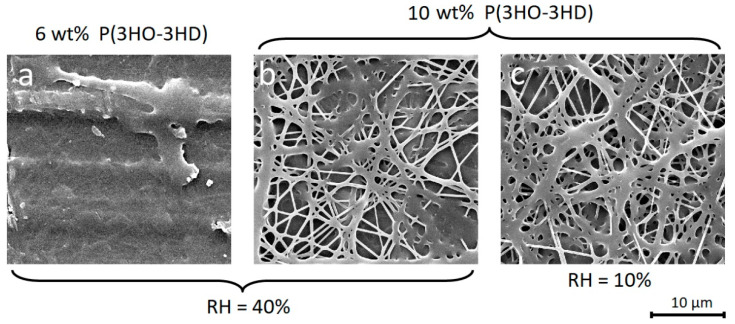
SEM images of P(3HO-*co*-3HD) nanofibers that were electrospun from a solution with 30% anisole, 35% acetone and 35% ether at 25 °C. While a sufficient polymer concentration is critical to avoid film formation by fused nanofibers (**a**,**b**), the effect of the relative humidity is less pronounced (**b**,**c**).

To circumvent carcinogenic halogenated solvents, P(3HO-*co*-3HD) nanofibers can be obtained from an eco-friendly solvent mixture that contains anisole, acetone and ether (Figure 2b,c). The electrospinning parameters for the nanofibers were investigated in terms of concentration, relative humidity (RH), temperature and solvent composition.

A sufficient polymer concentration is necessary to avoid the residual solvent fusing the fibers after deposition, and induce film formation (Figure 2a,b). The RH of the environment is deemed less critical, as similar fiber morphologies are observed at a RH of 10% and 40% (Figure 2b,c).

**Figure 3:**
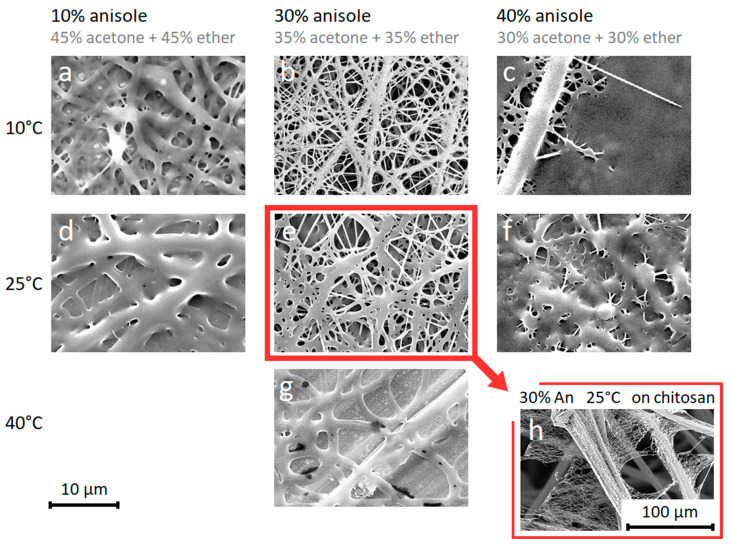
SEM images of 10% P(3HO-*co*-3HD) nanofibers with increasing anisole concentration from left to right. A lack of anisole (i.e., 10% anisole) (**a**,**d**) leads to too high evaporation rates resulting in thick fibers, while too much anisole (i.e., 40% anisole) (**c**,**f**) prevents sufficient evaporation resulting in fibers that are too wet, causing the polymer to fuse. When electrospinning at temperatures (i.e., 40 °C) too far above the polymer’s T_g_ (**g**), the polymer retains too much solvent after deposition resulting in thick fused fibers. The 30% anisole solutions at 10 °C (**e**) and 25 °C (**b**,**h**) result in a porous membrane at thin electrospinning layers, which can be deposited directly onto a chitosan carrier.

The electrospinning temperature does have a major effect on the fiber morphology, as an increase in temperature causes the fibers to become thicker due to the increased solvent evaporation rate that prevents the fibers stretching during the initial stages of electrospinning (Figure 3a,d). SEM images show that with electrospinning a 10 wt% P(3HO-*co*-3HD) solution in an anisole/acetone/ether (30%/35%/35%) mixture at 10 °C and RH of 40%, fine nanofibers are obtained with an average diameter of 300 nm (Figure 3b), while at 25 °C the fibers have a diameter of 440 nm (Figure 3e) and at 40 °C the fibers become 2 µm thick and fuse completely. The fusing of the fibers at higher temperatures can be attributed to the low specific fiber surface area of thick fibers, which increases drying times due to a reduced solvent evaporation rate. The polymer also approaches its melting temperature (57 °C), which additionally reduces its rate of crystallization/solidification (Figure 3g).

The importance of solvent composition was evaluated using 10% P(3HO-*co*-3HD) solution in three different anisole/acetone/ether solvent compositions (40%/30%/30%), (30%/35%/35%) and (10%/45%/45%) with a RH of 40%.

The (30%/35%/35%) anisole/acetone/ether mixture (Figure 3b,e) was found to be an optimum condition which gave more uniform nanofibers. The slow evaporating anisole needs to be balanced with the fast-evaporating acetone and ether, which is on the one hand because the electrospinning process requires an evaporation rate of the solution that is sufficiently high (Figure 3b,e) by limiting the anisole content to avoid a fused morphology (Figure 3c,f). On the other hand, the slow evaporating anisole prevents clogging at the nozzle tip and leads to a lower fiber diameter because the solvent allows the fibers to stretch more during the initial stages of the electrospinning process. These thin fibers (Figure 3b,e) have a higher specific fiber surface area that is in contact with the environment in comparison to solutions with less anisole (Figure 3a,d). The higher contact surface promotes solvent evaporation, both during electrospinning as well as after deposition onto the collector, hence avoiding fused fibers.

Although the neat P(3HO-*co*-HD) nanofibers become increasingly fused at longer electrospinning times (>30 min) at the same spot, preventing their applicability as a stand-alone nanofiber membrane, they could also be electrospun onto a chitosan carrier as a thin layer (Figure 3h). Thus, eco-friendly conditions can be preferred in cases where thin layers of neat P(3HO-*co*-3HD) nanofibers are sufficient. One of the main reasons for the fused morphology of the nanofibers at room temperature electrospinning is the low T_g_ of P(3HO-*co*-3HD). In order to eliminate this effect, a blend of P(3HB) and P(3HO-*co*-3HD) can be used, which is discussed in the subsequent subsection.

### 3.2. Blend Electrospinning of P(3HB)/P(3HO-co-3HD)

While standalone P(3HO-*co*-3HD) nanofibrous membranes cannot be electrospun at room temperature conditions and always need a backing carrier structure, the brittle P(3HB) has a proven track record of electrospinnability with halogenated solvents, avoiding fiber fusion altogether [26,54,55,56]. Nevertheless, P(3HB) has an elongation at break of less than 4%, making it too brittle for single usage as nanofibrous membranes in wound dressing applications. Hence, within this section a synergetic electrospinning mixture is aimed for by blend electrospinning to combine some of the flexibility of P(3HO-*co*-3HD) with the standalone and non-fused characteristics of P(3HB) nanofibers.

Ideally, a low toxicity solvent system is used for blend electrospinning P(3HB) and P(3HO-*co*-3HD). This requires the substitution of typical P(3HB) electrospinning solvents such as chloroform or dichloromethane. For this, we could turn to the literature on P(3HB) extraction (and PHA extraction in general) from bacterial cultures, which is, however, mostly based on the same chlorinated solvents [57,58,59]. To date, multiple attempts have been made to bypass these halogenated solvents for P(3HB) extraction from bacterial cultures and use less toxic solvents instead, such as butyl acetate, ethyl acetate [60], acetone/ethanol/propylene carbonate mixtures [61], anisole and cyclohexanone [62]. However, temperatures of 100 °C and above are always needed. Based on the literature review, we have tried various solvents and solvent mixtures for dissolving P(3HB) including benzyl alcohol, anisole, cyclohexanone, acetone, methyl ethyl ketone, dimethyl carbonate, ethyl acetate, their mixtures with anisole or benzyl alcohol, mixture of esters and alcohols, mixtures with DMSO and water. At 100 °C, the polymer could be dissolved in pure dimethyl carbonate, as well as solvent mixtures, including: ethanol/anisole (70%/30%), anisole/isopropanol/H_2_O (40%/50%/10%), anisole/DMSO (60%/40%) and dimethyl carbonate/DMSO (80%/20%). However, immediate solidification occurs at room temperature, making electrospinning (at room temperature) impossible.

Hence, for a successful electrospinning, a chlorinated solvent was included, and pure P(3HB) nanofibers were electrospun from a chloroform/2-butanol (70/30, *v*/*v*) mixture, which results in neat but brittle nanofibers with an average diameter of 775 ± 106 nm.

When electrospinning a blend of both polymers with the chloroform/2-butanol (70/30, *v*/*v*) solvent system, the ratio of P(3HB)/P(3HO-*co*-3HD) should be considered carefully as a high amount of P(3HB) can cause loss of elasticity, leading to brittle fibers, but a high P(3HO-*co*-3HD) may lead to fused fibers at large membrane thicknesses.

**Figure 4:**
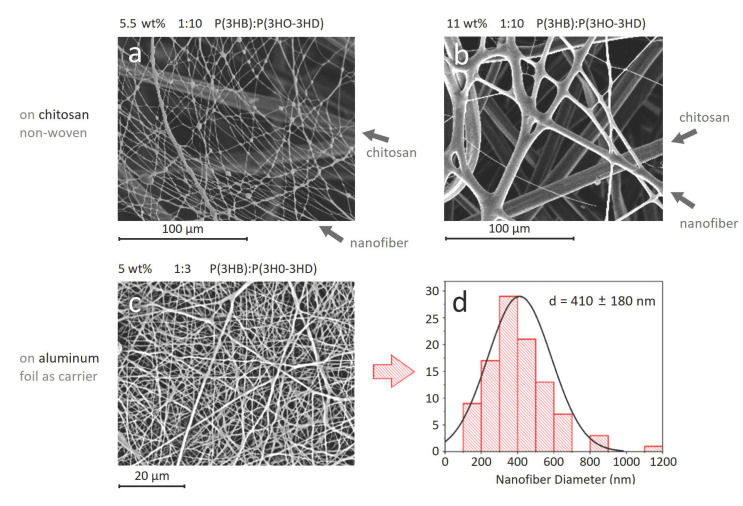
P(3HB)/P(3HO-co-HD) nanofibers with a concentration of (**a**) 5.5 wt%, (**b**) 11 wt% nanofibers, on chitosan carriers that are visible as thick fibers in the background. (**c**) SEM image and (**d**) histogram of the optimized P (3HB)/P(3HO-*co*-3HD) nanofibers.

Initially, the blend was electrospun from 5.5 wt and 11 wt% P(3HB)/P(3HO-*co*-3HD) (1/10, *w*/*w*) solutions onto chitosan carriers as wound dressing candidates, with average diameters of 470 ± 330 nm and 3420 ± 2100 nm, respectively (Figure 4a,b).

Optimization studies on the electrospinning of P(3HB)/P(3HO-*co*-3HD) blends were continued to obtain nanofibers that are as fine as possible while remaining flexible and bead-free. For this, a 5 wt% of P(3HB)/P(3HO-*co*-3HD) (1/3, *w*/*w*) was prepared in chloroform/2-butanol (70/30, *v*/*v*) (Figure 4c,d). The average diameter of the neat P(3HB)/P(3HO-*co*-3HD) nanofibers was 410 ± 180 nm with bead-free and homogenous morphology (Figure 4c). These nanofibers are used for AgNP immobilization in Section 3.3. The average diameter of nanofibers synthesized by different solution conditions are summarized in Table S1.

### 3.3. Modification and Characterization of the P(3HB)/P(3HO-co-3HD) Nanofibers with AgNPs

The modification and characterization processes were conducted on the P(3HB)/P(3HO-*co*-3HD) nanofibers based on the optimum 1/3 (*w*/*w*) blends. The P(3HB)/P(3HO-*co*-3HD) nanofibers were modified with silver nanoparticles (AgNPs) via a dip-coating technique to provide anti-microbial properties to the scaffolds. For this purpose, the nanofibers were first dipped into a water solution with colloidal silver (4000 ppm), containing particles in a size range of 10–80 nm, and subsequently taken out at a speed of 170 mm/min.

Three different protocols were examined in order to achieve the best silver deposition. In the first trial, P(3HB)/P(3HO-*co*-3HD) nanofibers were dipped in a beaker containing the colloidal silver solution and taken out immediately, followed by washing with distilled water and drying. In the second trial, the nanofibers were immersed into the aqueous silver solution and kept in the solution for 1 min. Afterwards, the samples were taken out, washed with distilled water and left to dry. In the last protocol, the nanofibers were dipped into the aqueous silver solution and taken out immediately for three successive times, followed by washing and drying.

**Figure 5:**
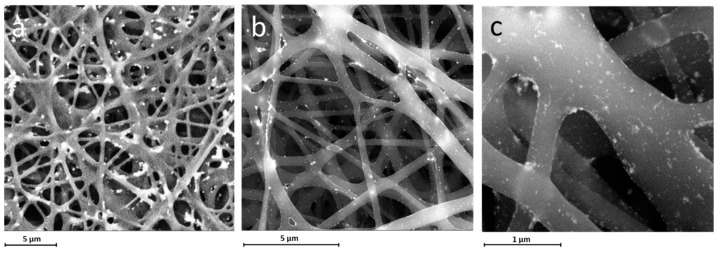
SEM images of AgNP-containing P(3HB)/P(3HO-*co*-3HD) nanofibers obtained by protocol 3 with magnifications (**a**) ×12,000, (**b**) ×25,000, (**c**) ×100,000 exhibit the uniform and dense immobilization of AgNPs on the nanofibers.

SEM images showed that the first protocol allowed the immobilization of the AgNPs. However, the distribution of the nanoparticles was not homogeneous and dense (Figure S4a). It was also observed that the second protocol did not contribute to achieving an intense and uniform dispersion of AgNPs over the P(3HB)/P(3HO-*co*-3HD) nanofibers (Figure S4b). Additionally, the nanoparticles had the tendency to agglomerate due to the increased immersion time. The most homogeneous and intense immobilization of the AgNPs was achieved using the third protocol. Although some minor agglomerations were still possible, a homogeneous dispersion of the silver nanoparticles over P(3HB)/P(3HO-*co*-3HD) nanofibers was clearly observed (Figure 5). Subsequently, the presence of silver nanoparticles on the P(3HB)/P(3HO-*co*-3HD) nanofibers was confirmed by EDX characterization and determined as 18 wt% and 5.3 atomic percentage, respectively.

FTIR-ATR spectra of neat P(3HB)/P(3HO-*co*-3HD) nanofibers and AgNP-loaded P(3HB)/P(3HO-*co*-3HD) nanofibers are given in Figure S3. There is no considerable shift of the peaks in the presence of silver nanoparticles. The spectrum between 3600 cm^−1^ and 3250 cm^−1^ represents the stretching band of the amine groups. The peaks at 2924 cm^−1^ and 2848 cm^−1^ can be attributed to asymmetric CH_3_ and symmetric CH_2_ vibrations, respectively. The peak at 1730 cm^−1^ is assigned to the ester carbonyl C = O stretching mode. In addition, the peak at 1279 cm^−1^ occurs because of C-N stretching and N-H bending. Moreover, as consistent with the literature, nearly the same spectrum was observed for AgNP containing P(3HB)/P(3HO-*co*-3HD) nanofibers [39,63,64].

**Figure 6:**
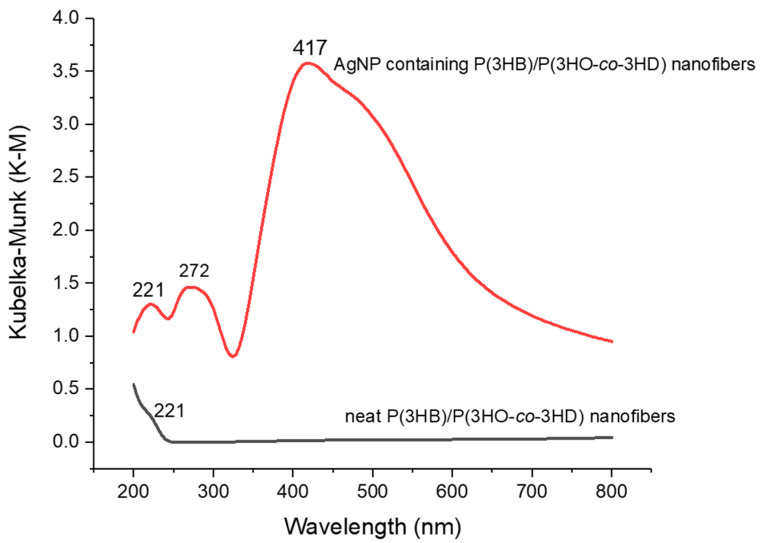
UV-Visible spectroscopy of neat P(3HB)/P(3HO-*co*-3HD) nanofibers (red) and AgNP containing P(3HB)/P(3HO-*co*-3HD) nanofibers (black). P(3HB)/P(3HO-*co*-3HD) nanofibers with AgNPs show a strong absorbance peak at 417 nm pointing out the AgNPs existence; however, neat P(3HB)/P(3HO-*co*-3HD) nanofibers do not show any peaks between 250 and 600 nm.

The AgNP deposition on the P(3HB)/P(3HO-*co*-3HD) nanofibers was also characterized by UV-Vis spectroscopy. While AgNP containing P(3HB)/P(3HO-*co*-3HD) nanofibers show a strong absorbance peak at 417 nm, neat P(3HB)/P(3HO-*co*-3HD) nanofibers do not show any peaks between 250–600 nm. This is in line with literature stating that the characteristic silver surface plasmon resonance band is attributed to the maxima between 400 and 420 nm, depending on the size of the particle, stabilizing molecules, and dielectric constant of the medium [39,46,64,65] and thus confirms the deposition of AgNPs on the nanofibers (Figure 6). The peak at 221 nm can be associated with the presence of ester groups in the polymer structure [66].

The biological activity of the AgNP-containing nanofibers was further examined. Alamar Blue assay was performed after a 24 h presence of HaCaT cell culture on the substrates. This shows that both the plain and the AgNP-containing P(3HB)/P(3HO-*co*-3HD) nanofibers did not show significant cytotoxic activity, which is in fact indicated by a dye reduction of 100% and 105%, respectively. Keratinocytes, which completely cover the outmost layer of our body, lead the immune reaction (i.e., innate immune response and inflammation) in the skin by the production of pro-inflammatory, anti-inflammatory and antimicrobial (i.e., defensins) molecules in response to injury and external stimulation. The complexity of immune reactions occurs via the production those cytokines and antibacterial molecules with a different time scale, which ultimately ensures an efficient tissue response to foreign agents.

**Figure 7:**
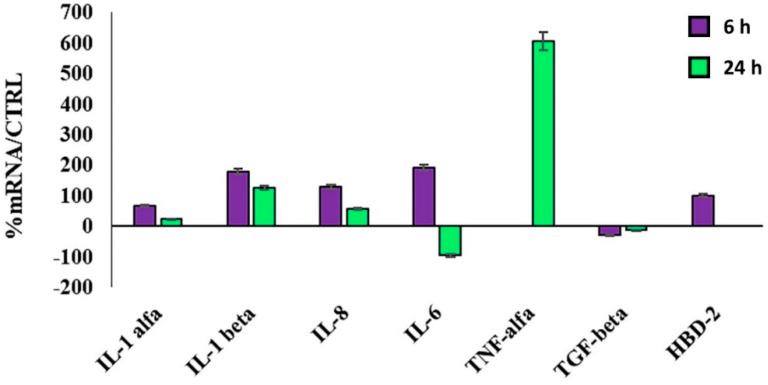
Relative gene expression of IL-1 (specifically, IL-1α and IL-1β), IL-6, IL-8, TNF-α, TGF-β by HaCaT cells in contact with P(3HB)/P(3HO-*co*-3HD)/Ag for 6 h and 24 h. Data are reported as mean ± SD and are expressed as percentage of increment relative to untreated HaCaT cells (as controls; CTRL).

Different from the plain P(3HB)/P(3HO-*co*-3HD) nanofibers, which did not show appreciable differences in the basal production of these molecules by HaCaT cells in culture, the AgNP-containing P(3HB)/P(3HO-*co*-3HD) nanofibers were able to upregulate the most important proinflammatory cytokines after 6 h, namely, IL-1(α and β), IL-6 and IL-8, in order to initiate the healing process (Figure 7). Later on, 24 h after being in contact with the cells, all the above-mentioned ILs are reduced. In contrast, TNF-α expression is only observed at 24 h. TNF-α is an essential mediator in inflammation by recruiting inflammatory cells [67]. It also acts on platelet adhesiveness, favoring the formation of thrombus to reduce bacterial invasion and infection processes. The modulation (namely, initial upregulation, followed by a downregulation) of pro-inflammatory cytokines can play a very useful role in wound healing. Indeed, the absence of proper healing has been attributed to a number of factors dealing with inefficient timing and extent of the inflammatory cascade [68,69]. Most importantly, the AgNP-containing P(3HB)/P(3HO-*co*-3HD) nanofibers can induce the mRNA production of HBD-2, thus stimulating the innate defense capability of the skin from harmful entities. This aspect is very relevant in the wound repair processes. In addition, such an indirect antimicrobial activity is desirable as the need for antibiotic drugs is reduced. The TGF β expression was insignificantly downregulated.

**Figure 8:**
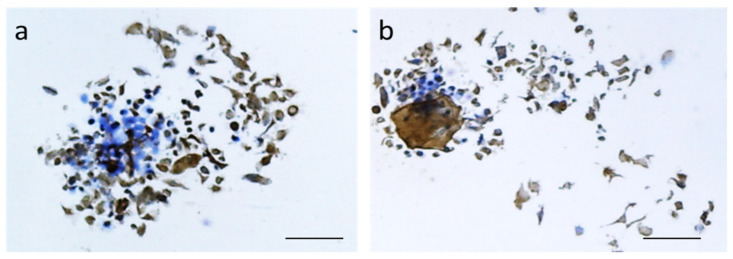
Immunocytochemistry for cytokeratin (revealed in brown) of HaCaT cells cultured for 7 days in: (**a**) unloaded and (**b**) Ag-loaded P(3HB)/P(3HO-*co*-3HD). Cell nuclei are stained in blue. Scale bar is 50 µm. Original magnification ×10.

In addition, HaCaT cells were able to express cytokeratin upon culture for 7 days onto the plain and Ag-loaded P(3HB)/P(3HO-*co*-3HD), as shown in Figure 8. This is a hall print of appropriate keratinocyte function, which is predictive of re-epithelization capacity [70]. In the presence of AgNPs, we could observe a dense expression of cytokeratin by HaCaT cells maintaining the squamous epithelial morphotype (Figure 8b) suggesting a good cytocompatibility.

## 4. Conclusions

The aim of this study was to produce wound dressing prototypes from bio-based polymers, namely PHAs. Only very thin layers of pure P(3HO-*co*-3HD) scaffolds could be prepared from the eco-friendly solvent system, as the fibers were fused due to the low T_g_ and elastomeric nature of the polymer. The morphology and elasticity of the nanofibers were optimized by blending P(3HO-*co*-3HD) with P(3HB), which has a higher T_g_ but lacks appropriate mechanical properties required for wound dressing applications due to its more brittle nature. Since P(3HB) had poor solubility in eco-friendly solvents, a chlorinated solvent-based system consisting of chloroform and 2-butanol was utilized for the electrospinning process, and P(3HB)/P(3HO-*co*-3HD) nanofibers with an average diameter of 410 ± 180 nm could be obtained. Afterwards, the surface of the fabricated P(3HB)/P(3HO-*co*-3HD) nanofibers was modified with AgNPs via a dip-coating method. The amount of AgNPs on the P(3HB)/P(3HO-*co*-3HD) nanofibers was determined as 18.05 wt% and 5.29 atomic % by EDX characterization. Metabolic activity, immunomodulatory, indirect antimicrobial activity and cytokeratin expression of both the modified nanofibers were examined in vitro using HaCaT cells, revealing the potential ability to effectively contribute to the wound healing process. In fact, also in presence of AgNPs, the metabolic activity of the cells was above 100%. Moreover, the most important ILs were modulated in 24 h, and HBD-2 was upregulated with respect to the basal condition of HaCaT cells specifically in Ag-loaded P(3HB)/P(3HO-*co*-3HD). Finally, a proper keratinocyte function was confirmed by the expression of cytokeratin at a protein level after 7 days in culture. These preliminary findings highlight the suitability of the produced AgNP-functionalized PHA-based nanofibers for biomedical applications, particularly as wound dressings.

## Figures and Tables

**Table 1:** The details of the quantitative reverse transcriptase polymer chain reaction (qRT-PCR). This includes operational conditions, gene, primer sequences, and product [32,33].

Molecule	Primer Sequence (Sense/Antisense)	Conditions	Size (bp)
IL-1 α	5′-CATGTCAAATTTCACTGCTTCATCC-3′ 5′-GTCTCTGAATCAGAAATCCTTCTATC-3′	5 s at 95 °C, 8 s at 55 °C, 17 s at 72 °C for 45 cycles	421
IL-1 β	5′-GCATCCAGCTACGAATCTCC-3′ 5′-CCACATTCAGCACAGGACTC-3′	5 s at 95 °C, 14 s at 58 °C, 28 s at 72 °C for 40 cycles	708
IL-6	5′-ATGAACTCCTTCTCCACAAGCGC-3′ 5′-GAAGAGCCCTCAGGCTGGACTG-3′	5 s at 95 °C, 13 s at 56 °C, 25′ s at 72 °C for 40 cycles	628
IL-8	5-ATGACTTCCAAGCTGGCCGTG-3′ 5-TGAATTCTCAGCCCTCTTCAAAAACTTCTC-3′	5 s at 94 °C, 6 s at 55 °C, 12 s at 72 °C for 40 cycles	297
TNF-α	5′-CAGAGGGAAGAGTTCCCCAG-3′ 5′-CCTTGGTCTGGTAGGAGACG-3′	5 s at 95 °C, 6 s at 57 °C, 13 s at 72 °C for 40 cycles	324
TGF-β	5′-CCGACTACTACGCCAAGGAGGTCAC-3′ 5′-AGGCCGGTTCATGCCATGAATGGTG-3′	5 s at 94 °C, 9 s at 60 °C, 18 s at 72 °C for 40 cycles	439
HBD-2	5′-GGATCCATGGGTATAGGCGATCCTGTTA-3′ 5′-AAGCTTCTCTGATGAGGGAGCCCTTTCT-3′	5 s at 94 °C, 6 s at 63 °C, 10 s at 72 °C for 50 cycles	198

## Data Availability

The raw data required to reproduce these findings cannot be shared at this time due to technical or time limitations but may be obtained from the authors upon reasonable request.

## Supplementary Materials

The following are available online at https://www.mdpi.com/article/10.3390/ma14174907/s1, Figure S1: Gas Chromatography Mass Spectrometry (GCMS) results of (a) P(3HB), (b) P(3HO-*co*-3HD), Figure S2: ^13^C Nuclear Magnetic Resonance (NMR) results of (a) P(3HB), (b) P(3HO-*co*-3HD), Figure S3: FTIR-ATR spectrum of neat P(3HB)/P(3HO-*co*-3HD) nanofibers (black) and AgNP-containing P(3HB)/P(3HO-*co*-3HD) nanofibers (red), Figure S4: SEM images of AgNP-containing P(3HB)/P(3HO-*co*-3HD) nanofibers obtained by (a) protocol 1, (b) protocol 2. Images with higher magnifications are given as insets.

## References

[1] Xie J., Li X., Xia Y. (2008). Putting electrospun nanofibers to work for biomedical research. Macromol. Rapid Commun..

[2] Li H., Wang M., Williams G.R., Wu J., Sun X., Lv Y., Zhu L.-M. (2016). Electrospun gelatin nanofibers loaded with vitamins A and E as antibacterial wound dressing materials. RSC Adv..

[3] Felgueiras H.P., Amorim M.T.P. (2017). Functionalization of electrospun polymeric wound dressings with antimicrobial peptides. Colloids Surf. B Biointerfaces.

[4] Zhang Y., Lim C.T., Ramakrishna S., Huang Z.-M. (2005). Recent development of polymer nanofibers for biomedical and biotechnological applications. J. Mater. Sci. Mater. Med..

[5] Bhardwaj N., Kundu S.C. (2010). Electrospinning: A fascinating fiber fabrication technique. Biotechnol. Adv..

[6] Greiner A., Wendorff J.H. (2007). Electrospinning: A fascinating method for the preparation of ultrathin fibers. Angew. Chem. Int. Ed..

[7] Pham Q.P., Sharma U., Mikos A.G. (2006). Electrospinning of polymeric nanofibers for tissue engineering applications: A review. Tissue Eng..

[8] Kim T.G., Park T.G. (2006). Surface functionalized electrospun biodegradable nanofibers for immobilization of bioactive molecules. Biotechnol. Prog..

[9] Liang D., Hsiao B.S., Chu B. (2007). Functional electrospun nanofibrous scaffolds for biomedical applications. Adv. Drug Deliv. Rev..

[10] Zhang Z., Hu J., Ma P.X. (2012). Nanofiber-based delivery of bioactive agents and stem cells to bone sites. Adv. Drug Deliv. Rev..

[11] Asghari F., Samiei M., Adibkia K., Akbarzadeh A., Davaran S. (2017). Biodegradable and biocompatible polymers for tissue engineering application: A review. Artif. Cells Nanomed. Biotechnol..

[12] Philip S., Keshavarz T., Roy I. (2007). Polyhydroxyalkanoates: Biodegradable polymers with a range of applications. J. Chem. Technol. Biotechnol..

[13] Keshavarz T., Roy I. (2010). Polyhydroxyalkanoates: Bioplastics with a green agenda. Curr. Opin. Microbiol..

[14] Akaraonye E., Keshavarz T., Roy I. (2010). Production of polyhydroxyalkanoates: The future green materials of choice. J. Chem. Technol. Biotechnol..

[15] Thomson N., Roy I., Summers D., Sivaniaha E. (2010). In vitro production of polyhydroxyalkanoates: Achievements and applications. J. Chem. Technol. Biotechnol..

[16] Masood F., Yasin T., Hameed A. (2015). Polyhydroxyalkanoates—What are the uses? Current challenges and perspectives. Crit. Rev. Biotechnol..

[17] Raza Z.A., Abid S., Banat I.M. (2018). Polyhydroxyalkanoates: Characteristics, production, recent developments and applications. Int. Biodeterior. Biodegrad..

[18] Nigmatullin R., Thomas P., Lukasiewicz B., Puthussery H., Roy I. (2015). Polyhydroxyalkanoates, a family of natural polymers, and their applications in drug delivery. J. Chem. Technol. Biotechnol..

[19] Brigham C., Sinskey A. (2012). Applications of polyhydroxyalkanoates in the medical industry. Int. J. Biotechnol. Wellness Ind..

[20] Chen G.Q., Zhang J. (2018). Microbial polyhydroxyalkanoates as medical implant biomaterials. Artif. Cells Nanomed. Biotechnol..

[21] Basnett P., Marcello E., Lukasiewicz B., Panchal B., Nigmatullin R., Knowles J.C., Roy I. (2018). Biosynthesis and characterization of a novel, biocompatible medium chain length polyhydroxyalkanoate by Pseudomonas mendocina CH50 using coconut oil as the carbon source. J. Mater. Sci. Mater. Med..

[22] Rai R., Keshavarz T., Roether J.A., Boccaccini A.R., Roy I. (2011). Medium chain length polyhydroxyalkanoates, promising new biomedical materials for the future. Mater. Sci. Eng. R Reports..

[23] Sudesh K., Lee Y.-F., Sridewi N., Ramanathan S. (2016). The influence of electrospinning parameters and drug loading on polyhydroxyalkanoate (PHA) nanofibers for drug delivery. Int. J. Biotechnol. Wellness Ind..

[24] Volova T., Goncharov D., Sukovatyi A., Shabanov A., Nikolaeva E., Shishatskaya E. (2014). Electrospinning of polyhydroxyalkanoate fibrous scaffolds: Effects on electrospinning parameters on structure and properties. J. Biomater. Sci. Polym. Ed..

[25] Grande D., Ramier J., Versace D.L., Renard E., Langlois V. (2017). Design of functionalized biodegradable PHA-based electrospun scaffolds meant for tissue engineering applications. N. Biotechnol..

[26] Masaeli E., Wieringa P.A., Morshed M., Nasr-Esfahani M.H., Sadri S., van Blitterswijk C.A., Moroni L. (2014). Peptide functionalized polyhydroxyalkanoate nanofibrous scaffolds enhance Schwann cells activity. Nanomed. NBM.

[27] Castro Mayorga J.L., Fabra Rovira M.J., Cabedo Mas L., Sánchez Moragas G., Lagarón Cabello J.M. (2018). Antimicrobial nanocomposites and electrospun coatings based on poly(3-hydroxybutyrate-co-3-hydroxyvalerate) and copper oxide nanoparticles for active packaging and coating applications. J. Appl. Polym. Sci..

[28] Li P., Zhang J., Liu J., Ma H., Liu J., Lie P., Wang Y., Liu G., Zeng H., Li Z. (2015). Promoting the recovery of injured liver with poly (3-hydroxybutyrate-co-3-hydroxyvalerate-co-3-hydroxyhexanoate) scaffolds loaded with umbilical cord-derived mesenchymal stem cells. Tissue Eng. Part A.

[29] Hawa A., Sudesh K., Sagadevan S., Mukheem A., Sridewi N. (2020). Physicochemical characteristics of poly(3-hydroxybutyrate) and poly(3-hydroxybutyrate-co-3-hydroxyhexanoate) electrospun nanofibres for the adsorption of phenol. J. Exp. Nanosci..

[30] Li W., Cicek N., Levin D.B., Liu S. (2019). Enabling electrospinning of medium-chain length polyhydroxyalkanoates (PHAs) by blending with short-chain length PHAs. Int. J. Polym. Mater. Polym. Biomater..

[31] Li W., Cicek N., Levin D.B., Logsetty S., Liu S. (2020). Bacteria-triggered release of a potent biocide from core-shell polyhydroxyalkanoate (PHA)-based nanofibers for wound dressing applications. J. Biomater. Sci. Polym. Ed..

[32] Azimi B., Thomas L., Fusco A., Kalaoglu-Altan O.I., Basnett P., Cinelli P., de Clerck K., Roy I., Donnarumma G., Coltelli M.B. (2020). Electrosprayed chitin nanofibril/electrospun polyhydroxyalkanoate fiber mesh as functional nonwoven for skin application. J. Funct. Biomater..

[33] Danti S., Azimi B., Candito M., Fusco A., Sajad S.B.M., Ricci C., Milazzo M., Cristallini C., Latifi M., Donnarumm G. (2020). Lithium niobate nanoparticles as biofunctional interface material for inner ear devices. Biointerphases.

[34] Mondal K., Sharma A. (2016). Recent advances in electrospun metal-oxide nanofiber based interfaces for electrochemical biosensing. RSC Adv..

[35] Krishnan P.D., Banas D., Durai R.D., Kabanov D., Hosnedlova B., Kepinska M., Fernandez C., Ruttkay-Nedecky B., Nguyen H.V., Farid A. (2020). Silver nanomaterials for wound dressing applications. Pharmaceutics.

[36] Dizaj S.M., Lotfipour F., Barzegar-Jalali M., Zarrintan M.H., Adibkia K. (2014). Antimicrobial activity of the metals and metal oxide nanoparticles. Mater. Sci. Eng. C.

[37] Choudhury H., Pandey M., Lim Y.Q., Low C.Y., Lee C.T., Marilyn T.C.L., Loh H.S., Lim Y.P., Lee C.F., Bhattamishra S.K. (2020). Silver nanoparticles: Advanced and promising technology in diabetic wound therapy. Mater. Sci. Eng. C.

[38] Destaye A.G., Lin C.K., Lee C.K. (2013). Glutaraldehyde vapor cross-linked nanofibrous PVA mat with in situ formed silver nanoparticles. ACS Appl. Mater. Interfaces.

[39] Castro-Mayorga J.L., Martínez-Abad A., Fabra M.J., Olivera C., Reis M., Lagarón J.M. (2014). Stabilization of antimicrobial silver nanoparticles by a polyhydroxyalkanoate obtained from mixed bacterial culture. Int. J. Biol. Macromol..

[40] Castro-Mayorga J.L., Fabra M.J., Lagaron J.M. (2016). Stabilized nanosilver based antimicrobial poly(3-hydroxybutyrate-co-3-hydroxyvalerate) nanocomposites of interest in active food packaging. Innov. Food Sci. Emerg. Technol..

[41] Castro-Mayorga J.L., Fabra M.J., Cabedo L., Lagaron J.M. (2017). On the use of the electrospinning coating technique to produce antimicrobial polyhydroxyalkanoate materials containing in situ-stabilized silver nanoparticles. Nanomaterials.

[42] Milazzo M., Gallone G., Marcello E., Mariniello M.D., Bruschini L., Roy I., Danti S. (2020). Biodegradable polymeric micro/nano-structures with intrinsic antifouling/antimicrobial properties: Relevance in damaged skin and other biomedical applications. J. Funct. Biomater..

[43] Danti S., D’Alessandro D., Mota C., Bruschini L., Berrettini S., Perale J., Hilborn G. (2017). Applications of bioresorbable polymers in skin and eardrum. Bioresorbable Polymers for Biomedical Applications: From Fundamentals to Translational Medicine.

[44] Mantovani A., Sica A., Sozzani S., Allavena P., Vecchi A., Locati M. (2004). The chemokine system in diverse forms of macrophage activation and polarization. Trends Immunol..

[45] Donnarumma G., Paoletti I., Fusco A., Perfetto B., Buommino E., de Gregorio V., Baroni A. (2016). β-defensins: Work in progress. Adv. Exp. Med. Biol..

[46] Sichani G.N., Morshed M., Amirnasr M., Abedi D. (2010). In situ preparation, electrospinning, and characterization of polyacrylonitrile nanofibers containing silver nanoparticles. J. Appl. Polym. Sci..

[47] Rujitanaroj P.O., Pimpha N., Supaphol P. (2010). Preparation, characterization, and antibacterial properties of electrospun polyacrylonitrile fibrous membranes containing silver nanoparticles. J. Appl. Polym. Sci..

[48] Karbownik I., Rac O., Fiedot M., Suchorska-Wozniak P., Teterycz H. (2015). In-situ preparation of silver-polyacrylonitrile nanocomposite fibers. Eur. Polym. J..

[49] Spadaro D., Barletta E., Barreca F., Curro G., Neri F. (2010). Synthesis of PMA stabilized silver nanoparticles by chemical reduction process under a two-step UV irradiation. Appl. Surf. Sci..

[50] Pastoriza-Santos I., Liz-Marzan L.M. (2000). Reduction of silver nanoparticles in DMF. Formation of monolayers and stable colloids. Pure App.Chem..

[51] Basnett P., Marcello E., Lukasiewicz B., Nigmatullin R., Paxinou A., Ahmad M.H., Gurumayum B., Roy I. (2020). Antimicrobial materials with lime oil and a poly(3-hydroxyalkanoate) produced via valorisation of sugar cane molasses. J. Funct. Biomater..

[52] Prat D., Wells A., Hayler J., Sneddon H., McElroy C.R., Abou-Shehada S., Dunn P.J. (2015). CHEM21 selection guide of classical- and less classical-solvents. Green Chem..

[53] Capello C., Fischer U., Hungerbühler K. (2007). What is a green solvent? A comprehensive framework for the environmental assessment of solvents. Green Chem..

[54] Xu X.Y., Li X.T., Peng S.W., Xiao J.F., Liu C., Fang G., Chen K.C., Chen G.Q. (2010). The behaviour of neural stem cells on polyhydroxyalkanoate nanofiber scaffolds. Biomaterials.

[55] Ramier J., Bouderlique T., Stoilova O., Manolova N., Rashkov I., Langlois V., Renard E., Albanese P., Grande D. (2014). Biocomposite scaffolds based on electrospun poly(3-hydroxybutyrate) nanofibers and electrosprayed hydroxyapatite nanoparticles for bone tissue engineering applications. Mater. Sci. Eng. C.

[56] Li X.T., Zhang Y., Chen G.Q. (2008). Nanofibrous polyhydroxyalkanoate matrices as cell growth supporting materials. Biomaterials.

[57] Jacquel N., Lo C.-W., Wu H.-S., Wei Y.-H., Wang S.S. (2007). Solubility of polyhydroxyalkanoates by experiment and thermodynamic correlations. AIChE J..

[58] Terada M., Marchessault R.H. (1999). Determination of solubility parameters for poly(3-hydroxyalkanoates). Int. J. Biol. Macromol..

[59] Ramsay J.A., Berger E., Voyer R., Chavarie C., Ramsay B.A. (1994). Extraction of poly-3-hydroxybutyrate using chlorinated solvents. Biotechnol. Tech..

[60] Aramvash A., Gholami-Banadkuki N., Moazzeni-Zavareh F., Hajizadeh-Turchi S. (2015). An environmentally friendly and efficient method for extraction of PHB biopolymer with non-halogenated solvents. J. Microbiol. Biotechnol..

[61] Fei T., Cazeneuve S., Wen Z., Wu L., Wang T. (2016). Effective recovery of poly-β-hydroxybutyrate (PHB) biopolymer from Cupriavidus necator using a novel and environmentally friendly solvent system. Biotechnol. Prog..

[62] Rosengart A., Cesário M.T., De Almeida M.C.M.D., Rodrigo S. (2015). Efficient P(3HB) extraction from *Burkholderia sacchari* cells using non-chlorinated solvents. Biochem. Eng. J..

[63] Gumel A.M., Annuar M.S.M., Ishak K.A., Ahmad N. (2014). Carbon nanofibers-poly-3-hydroxyalkanoates nanocomposite: Ultrasound-assisted dispersion and thermostructural properties. J. Nanomater..

[64] Phukon P., Saikia J.P., Konwar B.K. (2011). Enhancing the stability of colloidal silver nanoparticles using polyhydroxyalkanoates (PHA) from Bacillus circulans (MTCC 8167) isolated from crude oil contaminated soil. Colloids Surf. B Biointerfaces.

[65] Wang Y., Yang Q., Shan G., Wang C., Du J., Wang S., Li Y., Chen X., Jing X., Wei Y. (2005). Preparation of silver nanoparticles dispersed in polyacrylonitrile nanofiber film spun by electrospinning. Mater. Lett..

[66] Salgaonkar B.B., Mani K., Braganca J.M. (2013). Characterization of polyhydroxyalkanoates accumulated by a moderately halophilic salt pan isolate Bacillus megaterium strain H16. J. Appl. Microbiol..

[67] Esposito E., Cuzzocrea S. (2009). T NF-Alpha as a Therapeutic Target in Inflammatory Diseases, Ischemia- Reperfusion Injury and Trauma. Curr. Med. Chem..

[68] Azimi B., Maleki H., Zavagna L., De la Ossa J.G., Linari S., Lazzeri A., Danti S. (2020). Bio-Based Electrospun Fibers for Wound Healing. J. Funct. Biomater..

[69] Zhao R., Liang H., Clarke E., Jackson C., Xue M. (2016). Inflammation in Chronic Wounds. Int. J. Mol. Sci..

[70] Watanabe S., Osumi M., Ohnishi T., Ichikawa E., Takahashi H. (1995). Changes in cytokeratin expression in epidermal keratinocytes during wound healing. Histochem Cell Biol..

