# Supplementary material for: Silver Nanoparticle-Coated Polyhydroxyalkanoate Based Electrospun Fibers for Wound Dressing Applications
Source: Materials (Basel). 2021 Aug 28;14(17):4907. doi: 10.3390/ma14174907 (PMC8434271; doi:10.3390/ma14174907)
Supplement: Supplementary file 1 [file materials-14-04907-s001.zip › materials-1284964-supplementary.pdf]

materials

Supporting Information

Silver Nanoparticle-Coated Polyhydroxyalkanoate Based Electrospun Fibers for Wound Dressing Applications

Ozlem Ipek Kalaoglu-Altan [1,2], Havva Baskan [1,2], Timo Meireman [1], Pooja Basnett [3], Bahareh Azimi [4,5], Alessandra Fusco [4,6], Niccola Funel [7], Giovanna Donnarumma [4,6], Andrea Lazzeri [5], Ipsita Roy [8], Serena Danti [5] and Karen De Clerck [1,*]

[1] [1] Department of Materials, Textiles and Chemical Engineering, Ghent University, 9000 Ghent, Belgium; kalaoglualtan@itu.edu.tr (O.I.K.-A.); baskan@itu.edu.tr (H.B.); timo.meireman@ugent.be (T.M.), karen.de-clerck@ugent.be (K.D.C.),
[2] Department of Textile Engineering, Istanbul Technical University, 34437 Istanbul, Turkey
[3] School of Life Sciences, College of Liberal Arts and Sciences, University of Westminster, London W1W 6UW, UK; p.basnett@westminster.ac.uk
[4] Department/Laboratory, Interuniversity National Consortiums of Materials Science and Technology (INSTM), 50121 Firenze, Italy; bahareh.azimi@ing.unipi.it (B.A.); alessandra.fusco@unicampania.it (A.F.); giovanna.donnarumma@unicampania.it (G.D.)
[5] Department of Civil and Industrial Engineering, University of Pisa, 56126 Pisa, Italy; andrea.lazzeri@unipi.it (A.L.); serena.danti@unipi.it (S.D.)
[6] Department of Experimental Medicine, University of Campania "Luigi Vanvitelli", 80125 Naples, Italy
[7] Department of Translational Research and New Technologies in Medicine and Surgery, University of Pisa, 56126 Pisa, Italy; niccola.funel@gmail.com
[8] Faculty of Engineering, Department of Materials Science & Engineering, University of Sheffield, Sheffield S3 7HQ, UK; i.roy@sheffield.ac.uk
* Correspondence: karen.declerck@ugent.be

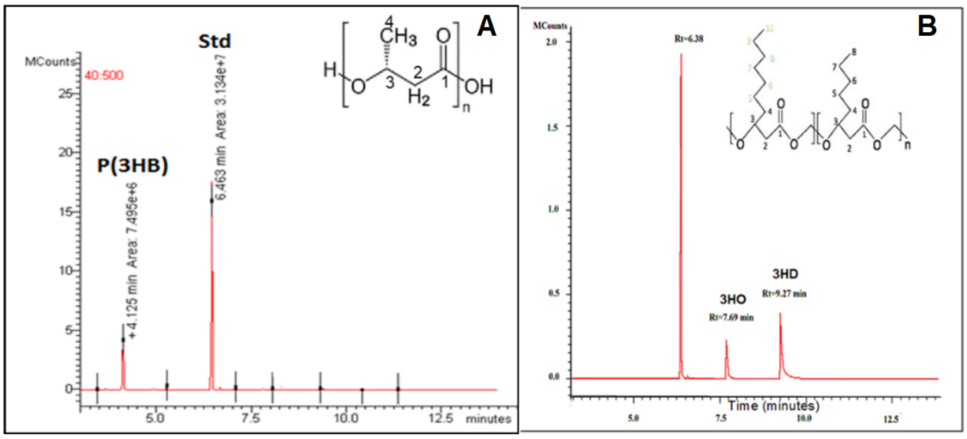

Figure S1. Gas Chromatography Mass Spectrometry (GCMS) results of (**A**) P(3HB), (**B**). P(3HO-*co*-3HD).

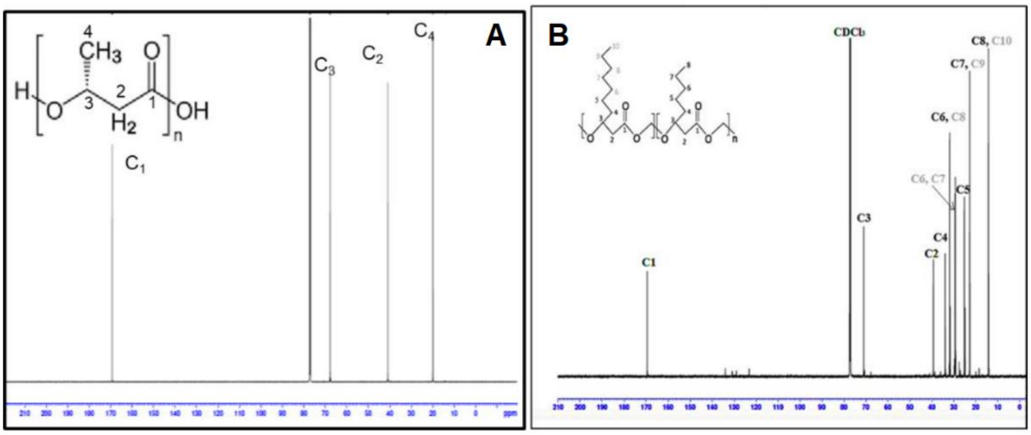

Figure S2. [13]C Nuclear Magnetic Resonance (NMR) results of (**A**) P(3HB), (**B**) P(3HO-*co*-.3HD).

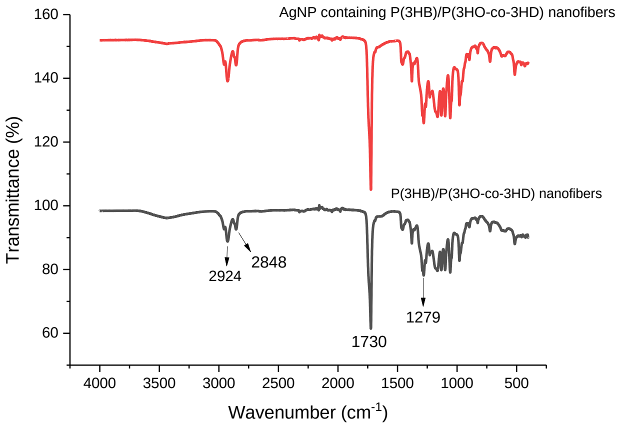

Figure S3. FTIR-ATR spectrum of neat P(3HB)/P(3HO-*co*-3HD) nanofibers (black) and Ag NPs containing P(3HB)/P(3HO-*co*-3HD) nanofibers (red).

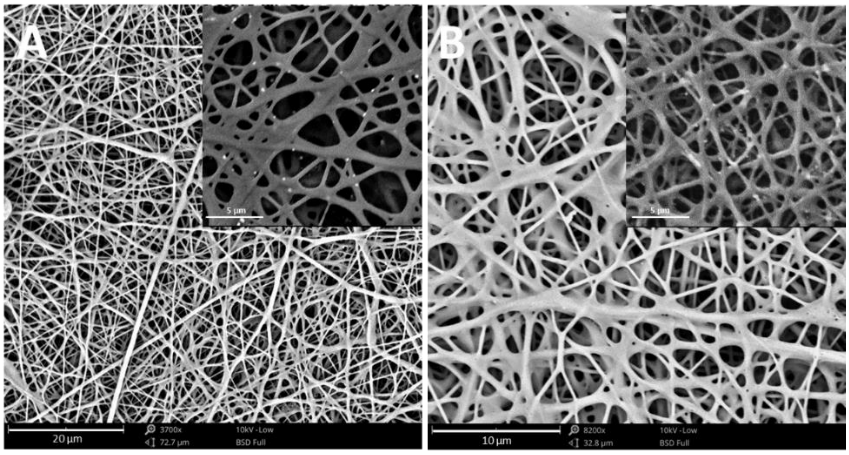

Figure S4. SEM images of AgNP-containing P(3HB)/P(3HO-*co*-3HD) nanofibers obtained by (A) protocol 1, (B) protocol 2. Images with higher magnifications are given as insets.
